# Biologically Inspired Stretchable, Multifunctional, and 3D Electronic Skin by Strain Visualization and Triboelectric Pressure Sensing

**DOI:** 10.1002/smsc.202100083

**Published:** 2021-11-05

**Authors:** Jing Li, Zuqing Yuan, Xun Han, Chunfeng Wang, Zhihao Huo, Qiuchun Lu, Meiling Xiong, Xiaole Ma, Wenchao Gao, Caofeng Pan

**Affiliations:** ^1^ Center on Nanoenergy Research School of Physical Science and Technology Guangxi University Nanning Guangxi 530004 P. R. China; ^2^ CAS Center for Excellence in Nanoscience Beijing Key Laboratory of Micro-nano Energy and Sensor Beijing Institute of Nanoenergy and Nanosystems Chinese Academy of Sciences Beijing 100083 P. R. China; ^3^ Department of Civil Engineering Monash University Clayton 3800 Australia; ^4^ School of Nanoscience and Technology University of Chinese Academy of Sciences Beijing 100049 P. R. China

**Keywords:** electronic skins, pressure sensing, strain visualization, 3D stress, triboelectric nanogenerators

## Abstract

Electronic skins have been developed for sensing vertical pressure and tensile strain individually. Here, a stretchable, multifunctional, and 3D electronic skin (SMTE) is fabricated to imitate the squid's natural structure to achieve strain visualization and pressure sensing at the same time. The SMTE consists of an anisotropic and elastic light shutter to tune the natural color or fluorescence, which converts the in‐plane strain to the visual changes in color and intensity. The shutter also works as an elastic dielectric layer for vertical pressure sensing based on the principle of a single‐electrode triboelectric nanogenerator. The performance of the SMTE is demonstrated by integrating it on a model hand as a tactile sensor for touch, bending, and gesture interpretation, which has potential applications in human–machine interaction, soft robots, and artificial intelligence.

## Graphical abstract

Inspired by the discoloration behavior of squids, a stretchable, multifunctional, and 3D electronic skin is designed to achieve strain visualization and pressure sensing. It converts the in‐plane strain into visual changes and the vertical pressure into electrical signals. This electronic skin has potential applications in human–machine interaction, soft robots, and artificial intelligence.

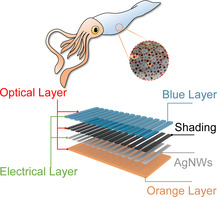

## 1 Introduction

Skin is one of the most sensitive and complicated sensory organs for humans and living bodies.^[^ ^1^ ^]^ It converts environmental stimuli into physiological signals, which nervous systems can further transport or interpret.^[^ ^2^ ^]^ Some species possess the extraordinary ability to change colors to gain advantages against prey or hide from potential predators.^[^ ^3^ ^]^ The bioinspired design has been an attractive strategy for electronic skins (E‐skins) to imitate the natural structure.[^3^, ^4^] Recently, E‐skins have been developed for sensing vertical pressure and tensile strains.^[^ ^5^ ^]^ However, the lack of 2D anisotropic tensile sensing properties has greatly limited their applications.

Triboelectric nanogenerators (TENGs) can generate electrical signals based on contact electrification and Maxwell displacement current.^[^ ^6^ ^]^ When TENGs work as self‐powered sensors, the output signals are correlated to the intensity of external stimuli. Furthermore, a wide range of flexible or stretchable materials can be adopted to manufacture TENGs and E‐skins.^[^ ^7^ ^]^ Lin et al. demonstrated a TENG sensor array for self‐powered static and dynamic pressure detection. Yang et al. developed a single‐electrode TENG for self‐powered sensing of human touch. Moreover, E‐skins can have the bioinspired function to change the color or luminance with stress or strain‐controlled microstructure for communication and camouflage.^[^ ^8^ ^]^ It is meaningful for E‐skins to develop other properties, such as the optical properties of tunable reflected color and fluorescence intensity.[^5^, ^8^]

In this work, a stretchable, multifunctional, and 3D electronic skin (SMTE) was fabricated to achieve strain visualization and vertical pressure sensing.^[^ ^9^ ^]^ The SMTE consisted of an elastic and stretchable shutter for the light transmission. The transverse stretching could open up the shutter to let light through and change the SMTE color.^[^ ^10^ ^]^ In contrast, the longitudinal stretching closed the shutter due to the Poisson's ratio and only changed the intensity of the original color.^[^ ^11^ ^]^ Another vertical pressure sensing was achieved by following the principle of single‐electrode TENG.[^9^, ^12^] The shutter also worked as an elastic dielectric layer for vertical pressure sensing. The electrical and optical performance of the SMTE was demonstrated by integrating it on a hand model. Such a combination of visual strain and electrical sensing is aimed to enhance the sensing performance as tactile sensors and effectively increases the information flux in a short time. The SMTE could work as a tactile sensor for touch, bending, and gesture interpretation, which has potential applications in human–machine interaction, soft robots, and artificial intelligence.[^8^, ^13^]

## 2 Results and Discussion

### 2.1 Concept and Working Principle of SMTE

**Figure 1:**
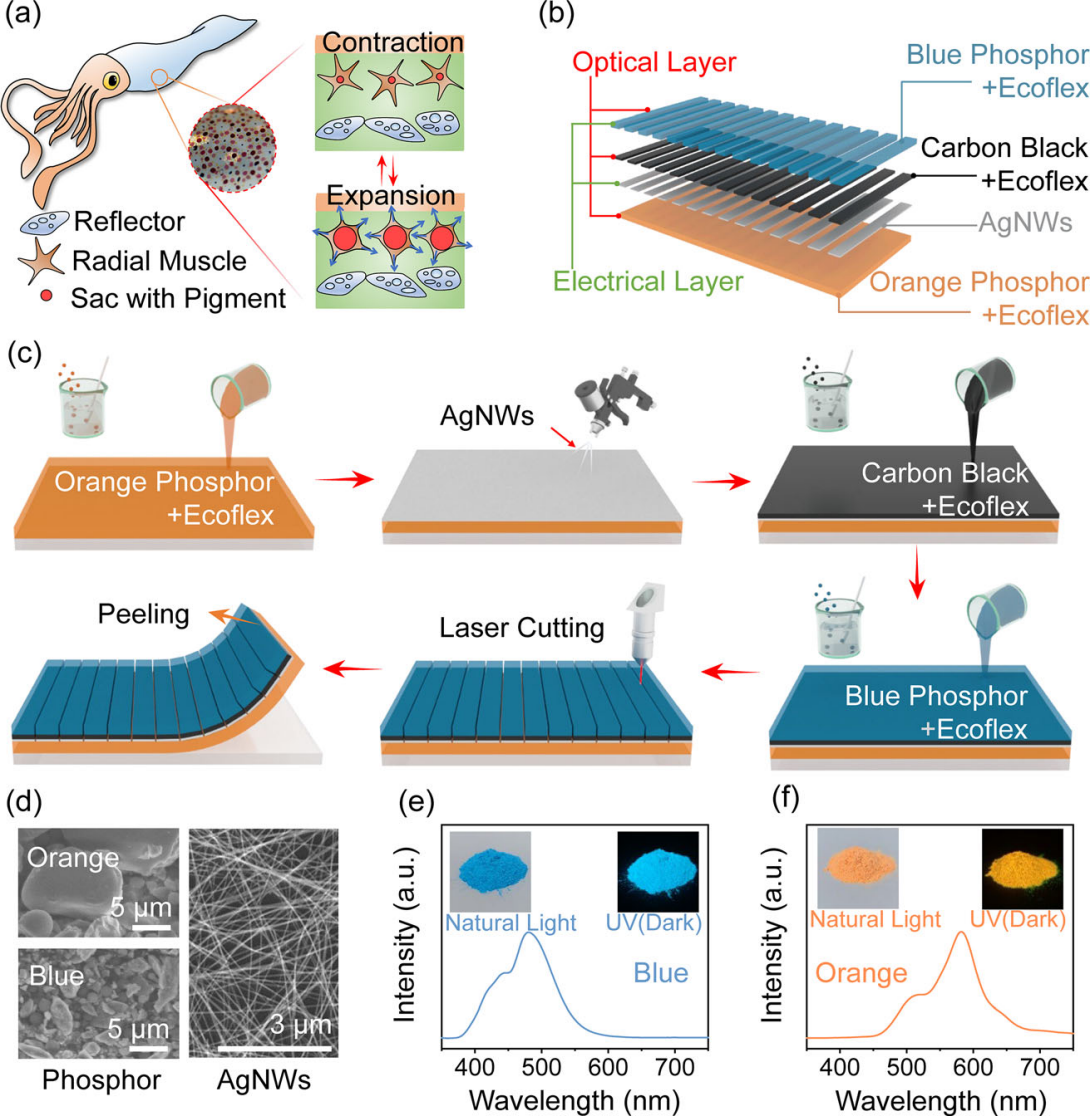
The design of the SMTE. a) Schematic diagram of the squid and its color‐change structure. b) Hierarchically schematic illustration of the SMTE on the enlarged functional area. c) The schematic illustration of the fabrication process of the SMTE. d) The SEM images of the phosphors and AgNWs. e,f) PL spectra of blue and orange phosphors.

Squids utilize the pigments to change the skin color for instant camouflage. **Figure** **1**a shows the squid and its epidermal cells. The pigment cells are in the capsule and only have an exposure area of 100 μm, which is almost invisible to the naked eye. Muscles are attached around them. When the muscle expands, the pigment cells rapidly expand to the millimeter range, displaying color patterns instantly. Inspired by this natural structure, the SMTE is designed to respond to tensile strains by anisotropic optical signals. The SMTE changes its color under transverse stretching and light intensity under longitudinal stretching.

The hierarchical structure of the SMTE on the enlarged functional area is shown in Figure 1b. The strontium aluminate phosphor doped with Eu and Dy is used as the visual material. The Ecoflex films are mixed with these phosphor powder to attain the corresponding color in the natural light. In addition, the phosphors also have fluorescence after UV irradiation. In the SMTE design, the orange layer is like the special pigment cells of squids, whereas the blue one is like the muscle to change the overall visualization. Between the orange and blue layers, another Ecoflex film is mixed with carbon black as a shading layer to prevent the light leak from the orange bottom layer. The shading layer is synchronously stretchable with the blue layer. Therefore, the orange layer bears the main stress under tensile strains, whereas there is a slight change in the blue and shading layers. During the expansion, the blue and shading layers work as the light shutter, and the orange layer finally changes the SMTE color. A silver nanowires (AgNWs) layer is fabricated under the blue layer as the electrode for the single‐electrode TENG. The fabrication process is shown in Figure 1c. First of all, the Ecoflex mixed with orange phosphors was spin coated on the precleaned acrylic plate to cure. Then AgNWs solution was sprayed on the orange layer, with conducting wires fixed for the following step. Another Ecoflex precursor mixed with carbon black was spin coated on the orange and AgNWs layers as a shading layer. Next, an Ecoflex layer mixed with blue phosphors was coated on the shading layer. After complete cure, the blue and carbon layers were cut by laser cutting to form regular and parallel cracks. The orange bottom layer was intact and exposed to external environments. Finally, the whole film was peeled off from the acrylic plate. The detailed fabrication process is in Experimental Section.

Figure 1d shows the scanning electron microscopy (SEM) images of the phosphor powder and AgNWs. The particle size of the orange phosphor was around 13 μm, while the blue phosphor was about 1.5 μm. Figure S1, Supporting Information, shows the X‐ray diffraction (XRD) data of the orange and blue phosphors, and the main component is strontium aluminate. The corresponding photoluminescence (PL) spectra of orange and blue phosphors were measured by an Edinburgh spectrometer, with the results in Figure 1e,f. The main PL peak of the blue phosphor was around 484 nm, and the main PL peak of the orange phosphor was around 584 nm. The photos of phosphors under the natural light and dark environment were in the insets, which the fluorescence in the dark was excited by a broadband UV lamp.

### 2.2 Visual Strain Sensing of the SMTE in the Transverse Direction

**Figure 2:**
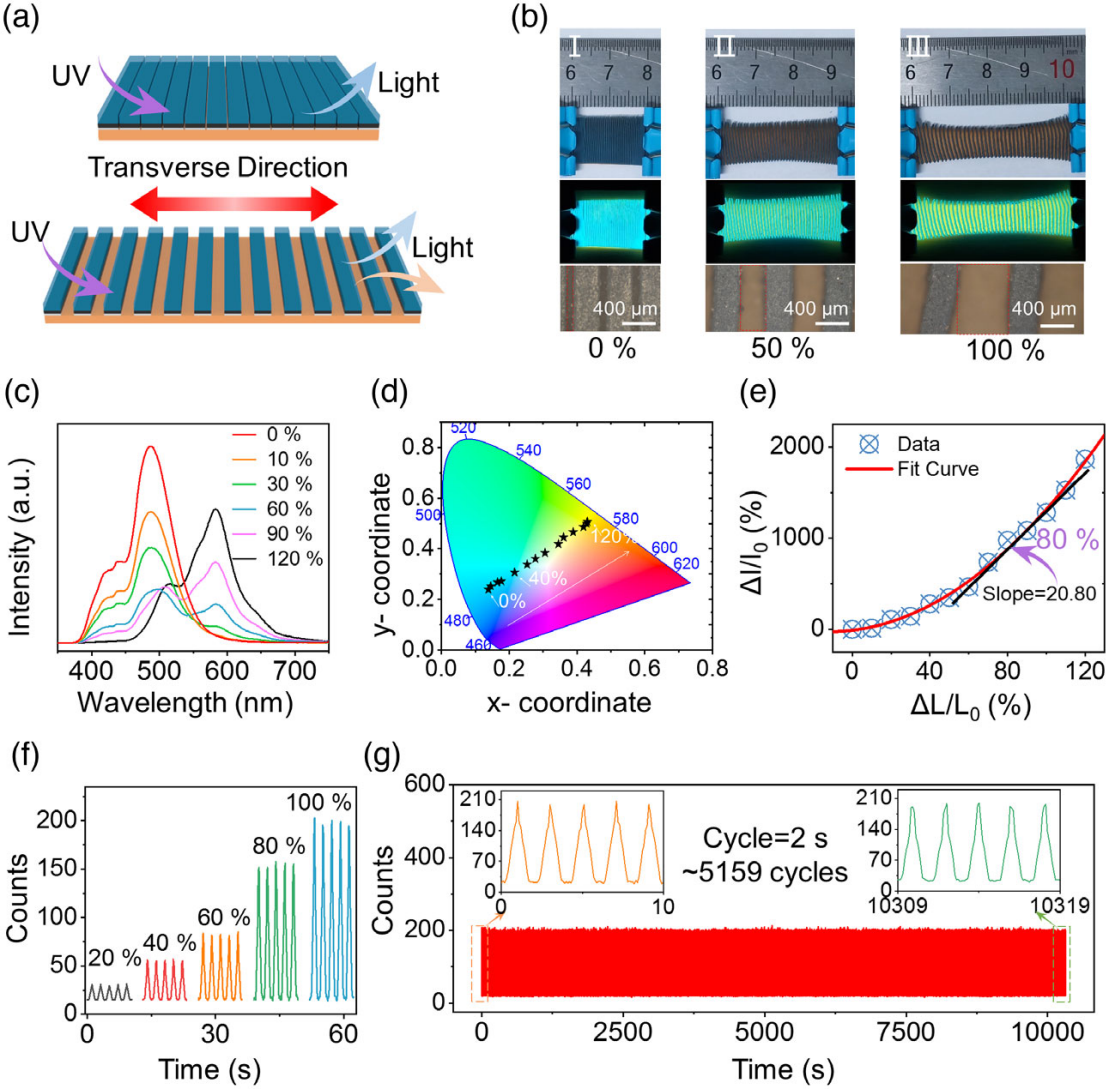
Visual strain sensing of the SMTE in the transverse direction. a) The working mechanism of the SMTE in the transverse direction to achieve visual color changes. b) The photos of the stretching SMTE under different tensile strains with the excitation from natural (top) and UV (middle) light. The microcracks were broadened under an optical microscope (bottom). c) The corresponding PL spectra of the SMTE under different tensile strains in the transverse direction. d) The CIE coordinates move from the blue to the orange region with increasing tensile strains. e) The 584 nm peak intensity of the PL spectra increased with the transversely tensile strains. f) The real‐time 584 nm intensity of the PL spectra in five stretching cycles under different transverse strains. g) Cycling tests of the SMTE to record the 584 nm peak intensity under 100% tensile strain (excited by the UV lamp).

SMTE was designed to achieve a highly sensitive and visual transverse stretching sensor, as shown in **Figure** **2**a. The SMTE had many parallel and periodic microcracks which were along the longitudinal direction. The cracks were precisely cut by the laser cutting, penetrating the upper layers to the orange bottom layer to attain a light shutter to regulate the size of the color area and the light leak from the orange bottom layer. When the SMTE was stretched transversely, the orange layer bore the main tensile stress, whereas there was little stress in the shutter. Therefore, the exposed area of the orange bottom layer increased gradually to emit corresponding fluorescence under environmental light excitation, realizing visual strain sensing. Figure 2b shows the photos of stretching SMTE under different tensile strains. The SMTE displayed its visual changes under natural and UV light. In the case of no stretching, the SMTE mainly emitted the blue glow from the upper layer. However, the width of microcracks increased gradually with the increasing tensile strain. The color changes could display the local stress distribution. The stretched Ecoflex film was wide on both ends and narrow in the middle due to the Poisson's ratio. The light shutter on the microscale was also observed under the optical microscope, as shown in the bottom part of Figure 2b. The microcracks changed from about 100 to 800 μm with increasing tensile strain, whereas the hills attained slight changes. Considering that the microcracks were uniformly fabricated in the transverse direction, the difference in the width resulted from the local stress distribution.

Figure 2c shows the fluorescent spectra of SMTE at different stretching strains of 0–120%. The detailed spectra of stretching strains with stepwise 10% increases were shown in Figure S2, Supporting Information. The corresponding spectra were attributed to the relative changes in the 484 and 584 nm peaks, which were adjusted by the light shutter. The peak intensity of 484 nm, mainly from the blue layer, decreased by 88%. On the contrary, the peak intensity of 584 nm, mainly from the orange layer, increased by 1866%. However, the orange layer also made contributions to the 484 nm peak. The 484 nm peak intensity no longer decreased when the orange peak reached saturation at the 120% tensile strain. In this situation, the width of the microcracks was too large to maintain the function of the light shutter to adjust the orange fluorescence.

The color changes could identify the transverse stretching strain of the SMTE. The coordinates in the commission internationale de L'eclairage (CIE) color space could describe the color changes under different transverse tensile strains, as shown in Figure 2d. In the measurement, the SMTE was excited by a stable 365 nm light to emit fluorescence. With the strain increasing, the coordinate gradually moved from the blue region to the orange region. The star markers in Figure 2d show the stepwise color changes from 0% to 120% stretching states. The results show that the color coordinates in the CIE diagram followed an approximately linear variation. Moreover, the changes of 584 nm intensity to strains were defined as the gauge factor for the transversely stretching SMTE, as shown in Figure 2e. There were sensitive visual responses to the continuously increasing strain. The gauge factor reached 20.8 at the 80% strain. Furthermore, the real‐time curves of the fluorescence intensity were measured during repetitive cycles of stretching. A portable spectrometer equipped with an optical fiber tested the fluorescence intensity of SMTE under tensile strains. The different strains could be distinguished by the intensity of the 584 nm peak, as shown in Figure 2f. The dynamic visual changes are shown in Video S1, Supporting Information. The SMTE had excellent sensing stability and reversibility in the transverse stretching process. The cycling test was carried out for the SMTE to record the real‐time curve of the 584 nm intensity. The tensile strain was set as 100%, and the period was 2 s in the experimental setup. The result is shown in Figure 2g. The light intensity varied between 15 counts for the release state and 203 counts for the 100% strain during the cycles, achieving the changes over 12 times. After more than 5000 cycles, the SMTE maintained the stable and sensitive sensing properties for tensile strain. The stable visual sensing was attributed to the elastic properties of the elastomer and the light shutter. The stretching strain below 200% was in the range of the elastic deformation of the SMTE. The measurement system to test the real‐time PL intensity of SMTE is shown in Figure S3, Supporting Information.

### 2.3 Visual Strain Sensing of the SMTE in the Longitudinal Direction

**Figure 3:**
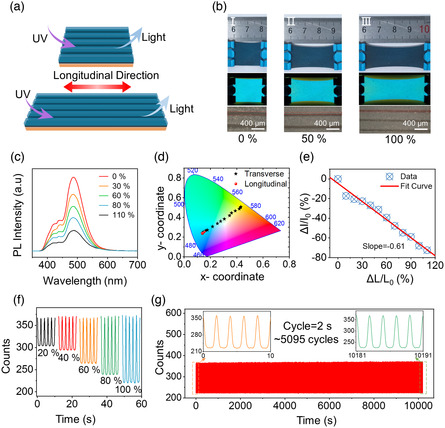
Visual strain sensing of the SMTE in the longitudinal direction. a) The working mechanism of the SMTE in the longitudinal direction to achieve visual strains. b) The photos of stretching the SMTE under different tensile strains with the excitation from natural (top) and UV (middle) light. The microcracks were closed under an optical microscope (bottom). c) The corresponding PL spectra of the SMTE under different longitudinal tensile strains. d) The CIE coordinates stayed in the blue region with increasing tensile strains labeled as the red markers. Only the intensity of the fluorescence changed. e) The 484 nm peak intensity of the PL spectra decreased with the tensile strains in the longitudinal direction. f) The real‐time 484 nm intensity in 5 stretching cycles under different longitudinal strains. g) Cycling tests of SMTE to record the 484 nm peak intensity under 100% tensile strain (excited by UV lamp).

To further discuss the anisotropic properties of in‐plane visual strain sensing, the SMTE performance was measured by stretching longitudinally. The schematic illustration is shown in **Figure** **3**a. The SMTE was stretched longitudinally, parallel to the microcrack direction, and measured its optical properties simultaneously. Under longitudinal tensile strain, the microcrack size decreased to close up. Therefore, the orange bottom layer could be hidden under the elastic shutter. Figure 3b shows the optical photos of different stretching states of SMTE under natural and UV light excitation. The SMTE remained to glow blue color even though the longitudinal strains increased from 0% to 100%. Simultaneously, the fluorescent intensity gradually decreased because the areal density of blue phosphor particles decreased. The bottom photos in Figure 3b show the microcracks under longitudinal strain tended to be closed due to the large Poisson's ratio of the elastic Ecoflex. Therefore, the orange bottom layer was hidden under the shutter.

The fluorescent spectra resulting from longitudinal tensile strains are shown in Figure 3c. The detailed spectra of the stretching states with a stepwise 10% strain increase were shown in Figure S4, Supporting Information. The main peak of the spectra was around 484 nm, indicating that the emitting fluorescence was mainly contributed to the blue top layer under longitudinal strains. The 484 nm peak intensity decreased by about 72% from 0% to 110% tensile strains because the tensile strains reduced the areal density of fluorescent particles. However, there were much smaller changes around the 584 nm peak. The results show that the longitudinal strain had little influence on the orange bottom layer, screened by the light shutter. The shading layer with carbon black prevented the excitation light from transmitting and screened the bottom fluorescence. Therefore, the CIE coordinates remained stable under longitudinal tensile strains, as shown in Figure 3d. The black stars are the coordinates of the transverse strains, and the red dots are the coordinate of the longitudinal stretching states. There was a distinguished difference between the two tensile conditions, which provided enough information to realize the in‐plane visual strain sensing. Angle tests for a square SMTE were carried out as stretching directions of 0°, 45°, 90°, 135°, and 180° to further discuss the in‐plane visual strain sensing. The visual changes displayed the strain distribution for this soft and stretchable device, as shown in Figure S5, Supporting Information.

The 484 nm peak intensity of the PL spectra decreased with the longitudinal tensile strains. Figure 3e shows the approximate linear curve of 484 nm peak intensity to the longitudinal stretching strains. The slope value of the curve was −0.61, as the longitudinal gauge factor of SMTE. Unlike the transverse sensing results, the fluorescent sensitivity of SMTE to longitudinal strain was related to the concentration of phosphor powder in the Ecoflex composite film or the areal density of phosphor particles. Moreover, the real‐time 484 nm peak intensity at different longitudinally tensile strains was measured. The detailed intensity curves of the tensile strains are shown in Figure 3f, displaying that SMTE had good reversibility and stability under longitudinal stretching. The SMTE was also tested for more than 5000 cycles to record the cycling changes in the fluorescence intensity, as shown in Figure 3g. The experiment was carried out under 100% strain and a 2 s period. During the stretching process, the 484 nm intensity was 380 counts at the release state and 221 counts at the 100% stretching strain. The light intensity waveform did not change significantly, indicating that SMTE had stable and long‐lasting sensing properties for longitudinal tensile strain.

### 2.4 Triboelectric Pressure Sensing of the SMTE in the Vertical Direction

**Figure 4:**
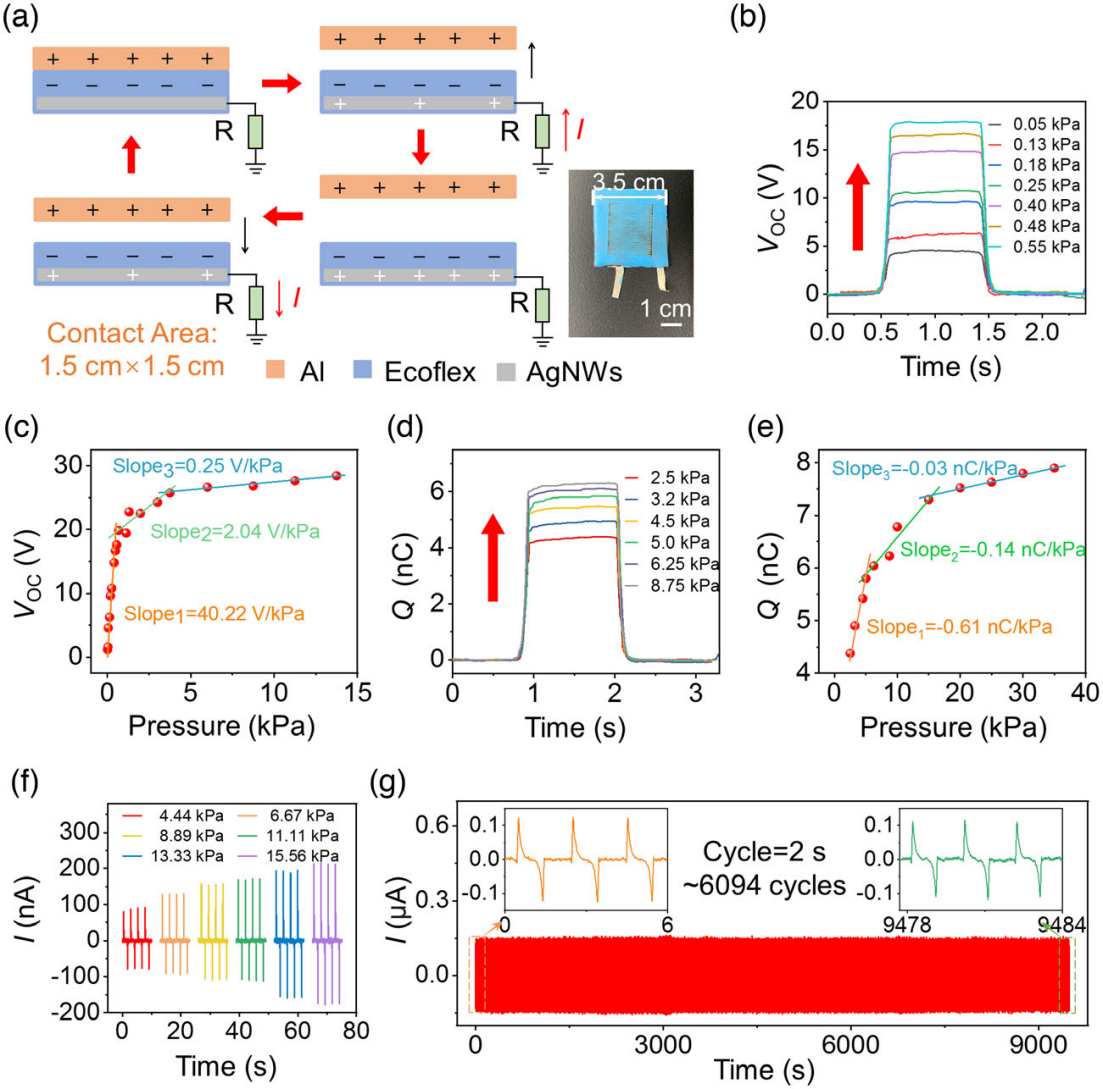
Electrical performance of the SMTE for vertical pressure sensing. a) The single‐electrode working principle of the SMTE as the vertical pressure sensing. The triboelectric device was shown in the inset. b,c) The open‐circuit voltage of the SMTE. b) The real‐time signals from 0.05 to 0.55 kPa; c) the curve of voltage to pressure in the range below 15 kPa. d,e) The output transfer charges of the SMTE. d) The real‐time signals from 2.5 to 8.75 kPa; e) the curve of transfer charges to pressure in the range below 40 kPa. f) The relationship between the short‐circuit current of the SMTE and vertical pressure. g) The cycling current test for the SMTE under 6.67 kPa pressure and 0.5 Hz frequency.

The microengineering structure usually works as the dielectric layer for capacitive and triboelectric sensors.^[^ ^14^ ^]^ The light shutter based on the microcracks also has pressure sensitivity in the vertical direction. In contrast, AgNWs could work as the flexible and stretchable electrode for the sensor because AgNWs conducting network has excellent conductivity and stability in the tensile state.^[^ ^15^ ^]^ The AgNWs were sprayed on the cured Ecoflex film and randomly oriented and evenly distributed to form a conductive network. The resistance of the AgNWs network was about 85 Ω before the laser cutting with a distance of 5 cm. Based on the microstructured Ecoflex and AgNWs electrode, the fabricated single‐electrode TENG could work as a self‐powered pressure sensor in the SMTE to transform the vertical pressure into electrical signals. **Figure** **4**a shows the working mechanism of the single‐electrode TENG in the SMTE. In the measurement setup, the light shutter contacted another Al film face to face. There was a charge transfer between the Al film and SMTE at the several initial contacts. The electrons in the Al film transferred onto the surface of Ecoflex due to the triboelectric series. When the Al film moved away from the Ecoflex, the electrostatic induction from the Al film was weakened. AgNWs gained electrons from the ground to maintain the overall electrostatic balance and became positively charged. Until the Al layer was farthest away from Ecoflex, the positive charges in AgNWs eventually reached an electrostatic balance with Ecoflex. There were several millimeters away to reach the highest open‐circuit voltage. When the Al film started to approach Ecoflex again, the positive charges in AgNWs decreased until the Al film contacted Ecoflex again.

The experiment tested the electrical performance of the SMTE for vertical pressure sensing. In this part, a measurement system was set up to characterize the electrical performance of the SMTE for vertical pressure sensing, as shown in Figure S6, Supporting Information. In the experimental setup, the SMTE was fixed on a flat plate and faced a dynamometer. The periodically vertical pressure is applied through a linear motor, whereas the electrical signals were recorded in real‐time by the data acquisition device. The size of the SMTE was 1.5 × 1.5 cm^2^ in the experiment. The relationship between the measured open‐circuit voltage (*V*_OC_) and vertical pressure is shown in Figure 4b,c. Figure 4b shows the real‐time pressure signals from 0.05 to 0.55 kPa. As the applied pressure increased, the *V*_OC_ increased. The generated voltage signals from the triboelectric pressure sensing were recorded by a real‐time monitor, as shown in Video S2, Supporting Information. Moreover, the electrostatic potential was maintained as the pressure load was on the SMTE. The voltage to pressure curve is shown in Figure 4c, by concluding the *V*_OC_ values in the pressure ranging from 0.05 to 13.75 kPa. Under 13.75 kPa, the *V*_OC_ increased to 28.3 V finally. In addition, the curve was mainly divided into three regions according to the different sensitivities in the large pressure region. In the first low‐pressure region (0.005–0.68 kPa), the SMTE had a higher‐pressure response sensitivity of 40.22 V kPa^−1^, and in the second medium‐pressure region (0.68–3.75 kPa), the sensitivity decreased to 2.04 V kPa^−1^. Finally, the sensitivity was as low as 0.25 V kPa^−1^ in the third high‐pressure region (3.75–13.75 kPa). The difference in sensitivity was mainly due to the states of contacting surface and the dielectric shutter between Al and Ecoflex. The Ecoflex and its grating structure had a small deformation at the low‐pressure region but apparent contact electrification and electrostatic induction, which achieved the highest sensitivity. While the pressure was in the medium region, there was an increase in the contact area and pressing strain. However, the electrical output of the SMTE was limited by the relatively small change in the contact area, which reduced the sensitivity to a certain extent. The dielectric layer was then more difficult to compress at the high‐pressure region, so the sensitivity became small. The contact area and compressive modulus were two competitive factors for the voltage signals. Compared with the microengineering SMTE, the flat‐surface device was tested to study the voltage to vertical pressure curve, as shown in Figure S7, Supporting Information. The highest *V*_OC_ reached 35 V due to the larger contact area. However, the sensitivity decreased in the low‐pressure region because of the larger compressive modulus. The medium and high pressure imposed the larger contact area to improve the sensitivity.

Similarly, the changes of transfer charges (*Q*) to the vertical pressure were measured. Figure 4d shows the corresponding *Q* in the pressure range of 2.5–8.75 kPa. The vertical pressure enhanced the output *Q* to over 6 nC at the pressure of 8.75 kPa. The various *Q* values were concluded in the pressure range from 2.5 to 35 kPa to draw a *Q* to pressure curve in Figure 4e. The curve was mainly divided into three pressure regions according to the different sensitivity. In the first low‐pressure region (lower than 4.9 kPa), the SMTE had a pressure sensitivity of 0.61 nC kPa^−1^. The sensitivity decreased to 0.14 nC kPa^−1^ in the medium‐pressure region (4.9–15.2 kPa), further decreasing to 0.03 nC kPa^−1^ in the third high‐pressure region (15.2–35.1 kPa). Moreover, the output short‐circuit current (*I*) was related to the vertical pressure. There was a current increase under larger vertical pressure, as shown in Figure 4f. Different from the voltage, the current followed a dynamic mechanical response. The cycling test for the current characterized the electrical stability and durability of the SMTE. The device was set under 6.67 kPa pressure and tested for over 6000 cycles, as shown in Figure 4g, displaying excellent electrical stability.

### 2.5 Demonstration as Finger SMTEs

**Figure 5:**
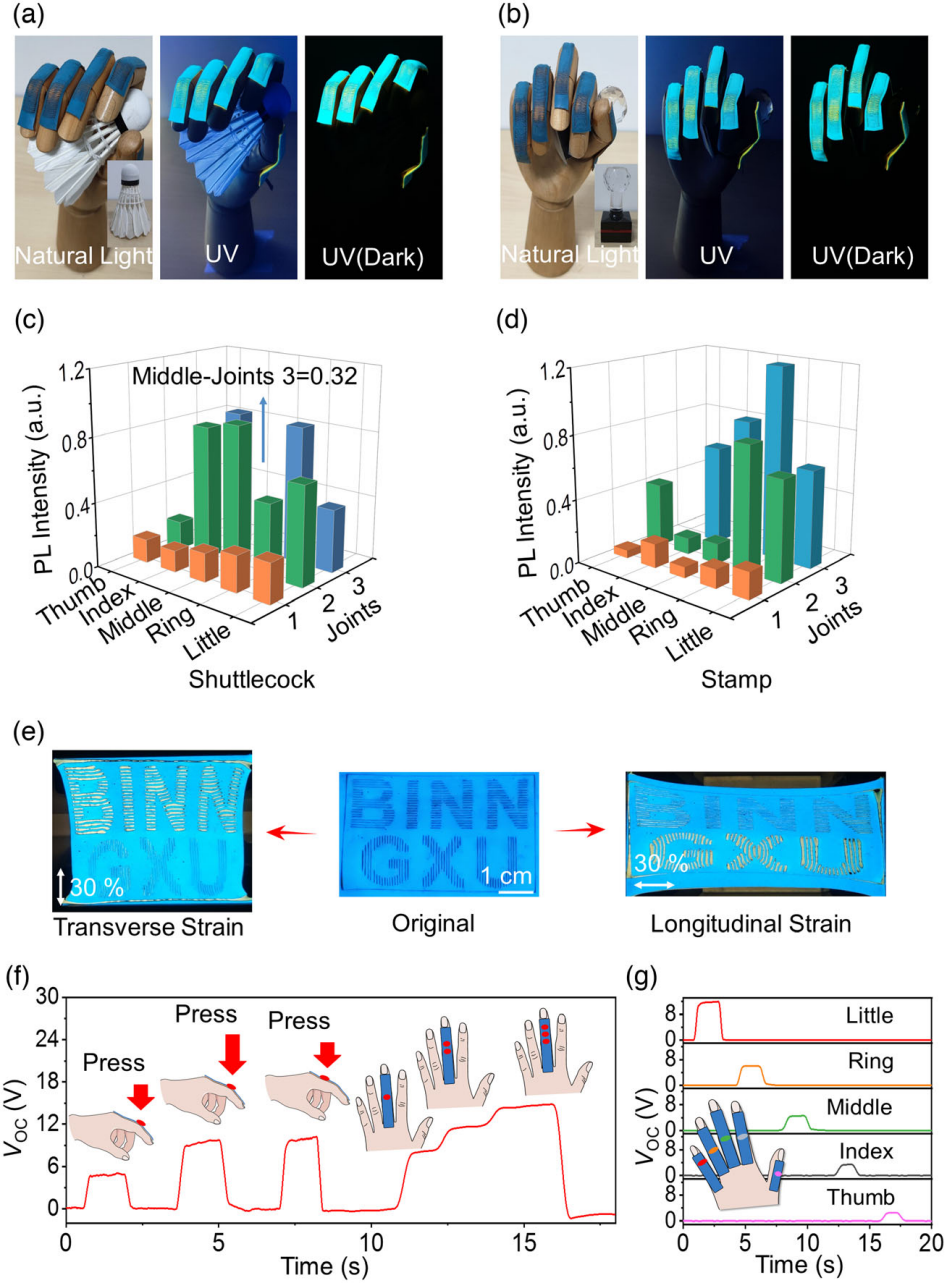
The electrical and visual performance of the finger SMTEs. a,b) Photos of the finger SMTEs to display the strain distribution based on the color change. Two gestures were tested to grasp the a) shuttlecock and b) stamp under the natural light, UV light, and UV (full dark) environments. The insets at the left bottom corners were the corresponding objects. c,d) The ratio of the PL intensity of the finger SMTEs at 584 and 484 nm when grasping the c) shuttlecock and d) stamp. The light signals were collected from three joints of the fingers except for the thumb with two joints. e) Photos of “BINN, GXU” on the SMTE in transverse and longitudinal tensile directions. f) Open‐circuit voltage of a finger SMTE in the conditions of different pressure, different locations, and large effective working regions. g) Open‐circuit voltage from the finger SMTEs on five fingers.

The SMTE worked based on the in‐plane visual strain sensing and vertical pressure sensing. The SMTEs were attached on the fingers of a hand model. The hand model was utilized to grasp a shuttlecock and a stamp, with different gestures shown in **Figure** **5**a,b. The bending finger joints drove the SMTEs, of which the deformation could be observed in the natural light, UV, and UV(dark) conditions. Video S3, Supporting Information, shows the bending finger SMTE that was excited by UV light. As the strain increased, the orange bottom layer was exposed to the environment. The SMTE color was related to the bending degree of the joint, which was described in Figure S8, Supporting Information. The spectra of finger SMTEs were close measured by a portable spectrometer with an extended optical fiber. There were three joints at each finger of the hand model except for the thumb with two joints. The SMTE could reflect the local strain that was induced by the bending joints. The visual strain changes could be observed in the Video S3, Supporting Information. Figure 5c,d shows the visual strain distribution of two different gestures, which was described by the peak intensity ratio of 584 to 484 nm at the joints. The gestures of grasping shuttlecock and stamp were translated into different optical signals. The results in Figure 5a–d show that the SMTE could quantitatively display the local strains in visual changes, which was easy to recognize by human eyes or cameras instead of lots of wiring and power units. Furthermore, SMTE was based on a highly reversible and stable structure to achieve visual changes. These visual changes were controllable by the microcracks and composite, which could display its characteristics under natural and UV light. An overview of the color distribution or the detailed postprocess could easily recognize the gesture. Furthermore, the SMTE could be utilized for display optics. The experiment utilized a “BINN, GXU” patterned SMTE to describe the anisotropic properties. The “BINN” were composed of parallel microcracks in the longitudinal direction, whereas the microcracks of “GXU” were in the transverse direction. When stretching from the original shape, different orientations induced the color changes of the letters, as shown in Figure 5e. As the manufacture methods of microcracks and color composites were controllable, the SMTE could be fabricated into complex outlines and shapes, which expanded its usage scenarios.

The finger SMTE could also respond to external pressure while maintaining the visual strain sensing. Figure 5f shows the open‐circuit voltage of a finger SMTE in the conditions of different pressure, different locations, and large effective working regions. The pressure was applied by finger touch. Under relatively light pressure, the output voltage of the SMTE was about 5.2 V, and it increased to about 9.8 V under a larger pressure. Similarly, the output voltage at different parts of the bending finger SMTE was measured. The simultaneous pressure at several locations could also enhance the voltage output. However, the variation of voltage was usually related to the contact area. For example, there was a difference in voltage signals between the joint and phalanx, which should be standardized to precisely sense in different conditions. Moreover, the SMTE could work as an electrically tactile array, as shown in Figure 5g. Each finger SMTE could work independently to sense the pressure. The experiment tested the voltage signals at joint 2 when the fingers were bending. The bending degrees in different fingers influenced the output voltage. The voltage that could be read out at the thumb was about 11.8 V and was about 2.51 V at the little finger. In this way, SMTE could sense small touches, demonstrating the coexist of self‐powered electrical and visual sensing and supplying more tactile information. Another Supporting Information, Video S4, displayed that the external pressure had little influence on the visual strain of the SMTE supported by a hard substrate. In a word, the SMTE had potential applications for smart skins, human–machine interaction, and prostheses.

## 3 Conclusion

In this work, a stretchable, multifunctional, and 3D E‐skin is fabricated to achieve in‐plane strain visualization and vertical pressure sensing. The visual in‐plane strain sensing part consists of fluorescent elastomer films and a light shutter with many parallel microcracks. Induced by the external force, the size of microcracks controls the transmittance of the bottom fluorescence to display the local strain distribution. The microcracks open up in the transverse direction while closing up in the longitudinal direction due to the Poisson's ratio. Therefore, the light shutter can distinguish the transversely and longitudinally tensile strains. Compared with the rigid film with polyvinyl alcohol (PVA) and other materials, the microcracks in the SMTE can be controlled precisely by the microengineering process. By developing optimum cracks, SMTE can perform transversely tensile sensing in the strain range of 0–120% with an intensity sensitivity up to 20.8. The results show that the color coordinates in the CIE diagram followed an approximately linear variation from the blue region to the orange region. SMTE realizes the longitudinal tensile sensing in the strain range of 0–100%, with the fluorescence intensity decreasing over 60%. Moreover, the fabricated SMTE can realize sensitive and stable visual in‐plane strain sensing.

The vertical pressure part of the SMTE is based on the single‐electrode mode TENG. A stretchable conductive network of AgNWs is embedded in the medium layer, and the elastomer shutter works as the triboelectric and dielectric layer. The open‐circuit voltage can reach 28.3 V, and the transfer charges can reach 6 nC. The SMTE achieves a high sensitivity up to 40.22 V kPa^−1^ or 0.61 nC kPa^−1^ for vertical pressure sensing. The fabricated triboelectric part can work stably for long cyclings.

Finally, the finger SMTEs are attached to the hand model to demonstrate its 3D sensing of external stimuli. The finger SMTEs are appropriate to the finger shape and can respond to the model gestures with corresponding visual and electrical signal outputs. Moreover, the multidimensional properties of the finger SMTE are demonstrated by the anisotropic letter shapes. In conclusion, the SMTE has potential applications in human–machine interaction, soft robotics, and artificial intelligence.

## 4 Experimental Section

4.1

4.1.1

##### Fabrication Process of the SMTE

First, the fluorescent films were fabricated with silicone rubber (Smooth‐on, Ecoflex 00‐10) and phosphor powder (strontium aluminate: Eu^2+^, Dy^3+^). The orange fluorescent film was prepared by mixing the Ecoflex precursor and orange phosphor powder in a 3:1 weight ratio, then pouring onto an acrylic plate. The area of the acrylic plate was precleaned and enclosed with a rectangle mold. The film was about 400 μm thick, cured at room temperature for at least five hours. Second, the AgNW solution was sprayed by an airbrush (Master Airbrush G444‐SET, 0.5 mm nozzle) to form a AgNW conductive network on the orange fluorescent films. Other conducting tapes were attached to the AgNW network to collect the electrical signals. The 50 nm‐diameter AgNWs of 20 mg mL^−1^ in water were bought from m Nanjing XFNANO Materials Tech Co., Ltd. Third, the Ecoflex precursor mixed with carbon black powder in an 8:1 weight ratio was spin coating onto the AgNW network. The carbon black layer was about 200 μm thick and cured at room temperature for at least 5 h. Next, the Ecoflex precursor with blue phosphor powder in a 3:1 weight ratio was spinning onto the carbon black layer. The blue fluorescent layer was 300 μm thick and cured at room temperature for at least 5 h. Finally, a laser cutting machine was utilized to cut the parallel microcracks that penetrate the blue fluorescent layer and carbon black layer until the orange bottom layer. The fabricated SMTE could be peeled from the acrylic plate.

##### Optical and Electrical Measurements

The stable PL spectra of SMTE under various strains were measured on a spectrometer (Edinburgh FLS 1000) with a slit width of 1.5 nm. The dynamic curve of PL intensity to time was measured by a portable spectrometer (QE65‐PRO) with a homemade motorized positioning system. The open‐circuit voltage, short‐circuit current, and transfer charges of SMTE were measured using an electrometer (Keithley 6514) with the help of a dynamometer (ATI nano17) and linear motor (Linmot). The optical photos of microcracks were captured by an optical microscope (Zeiss Scope A1). The XRD data were measured by an X‐ray diffractometer (PANalytical, X′Pert 3 Powder) with a Cu Kα radiation source. The morphology of phosphor powder was captured by an SEM (Hitachi, SU1510).

## Conflict of Interest

The authors declare no conflict of interest.

## Data Availability Statement

Research data are not shared.

## Supporting information

Supplementary Material
