# Supplementary material for: Biologically Inspired Stretchable, Multifunctional, and 3D Electronic Skin by Strain Visualization and Triboelectric Pressure Sensing
Source: Small Sci. 2021 Nov 5;2(1):2100083. doi: 10.1002/smsc.202100083 (PMC11935989; doi:10.1002/smsc.202100083)
Supplement: Supplementary file 1 — Supplementary Material [file SMSC-2-2100083-s001.zip › Supporting Information.pdf]

Supporting Information

Biologically Inspired Stretchable, Multifunctional and Three-Dimension Electronic Skin by Strain Visualization and Triboelectric Pressure Sensing

J. Li, Z. Yuan, X. Han, C. Wang, Z. Huo, Q. Lu, M. Xiong, X. Ma, W. Gao*, C. Pan**

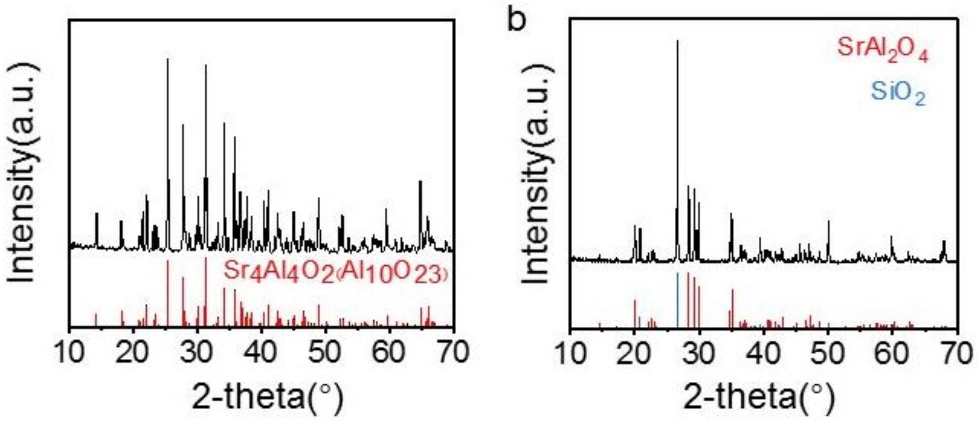

Figure S1. The XRD data of the blue (a) and orange (b) phosphor powder.

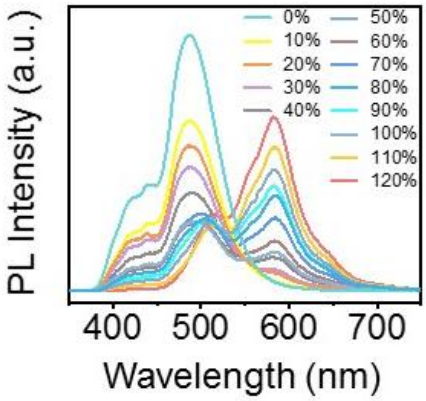

Figure S2. The PL spectra change to the transversely tensile strains with stepwise 10 % increases.

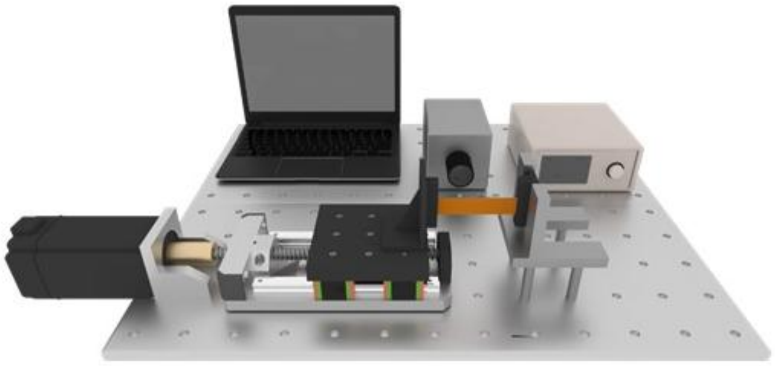

Figure S3. The measurement setup of the real-time visual strain sensing in the transverse and longitudinal directions.

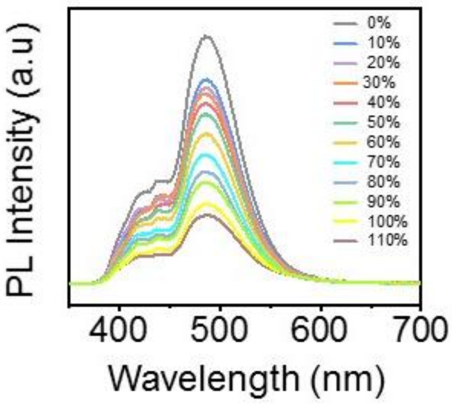

Figure S4. The PL spectra change to the longitudinally tensile strains with stepwise 10 % increases.

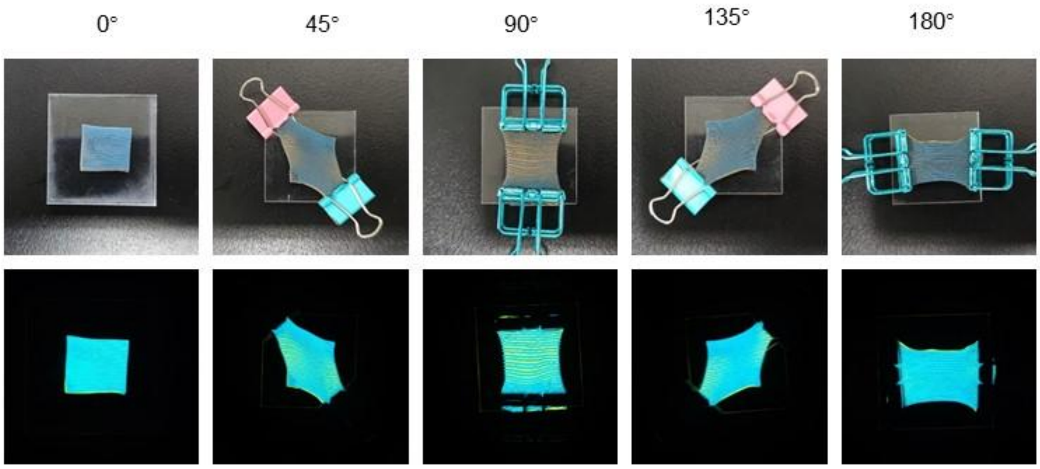

Figure S5. The in-plane angle tests for a square SMTE as stretching directions of 0, 45, 90, 135, and 180 degrees to display visual strains.

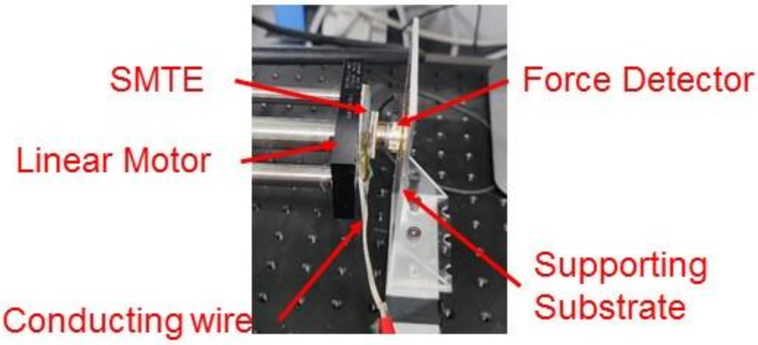

Figure S6. The measurement setup of the triboelectric vertical pressure sensing.

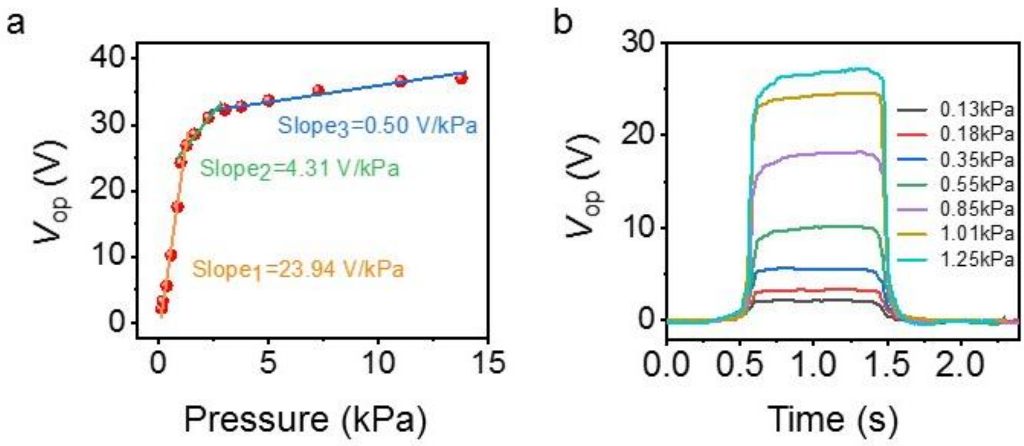

Figure S7. The electrical performance of the single-electrode TENG with a flat film.

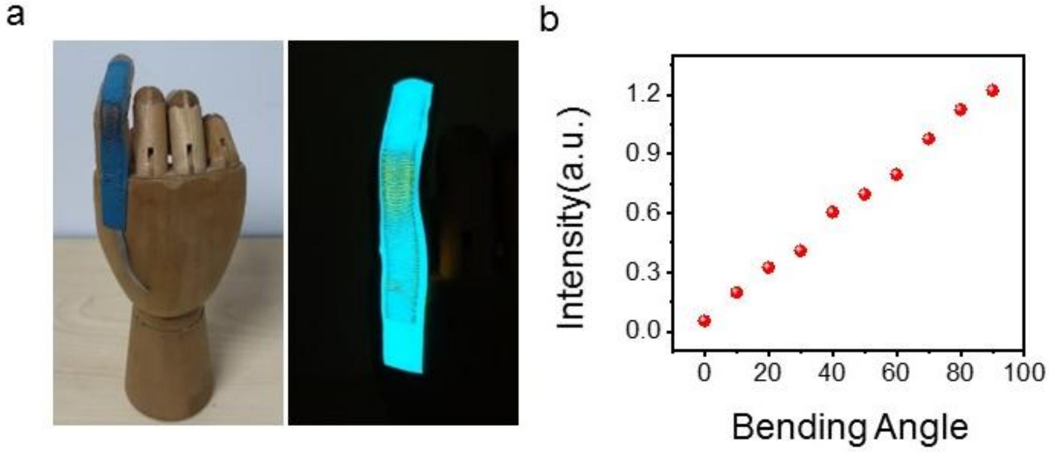

Figure S8. a. The photos of bending finger SMTE under the natural and dark environment. b. The SMTE intensity responded to the bending angle.
